# Experience-Dependent Rewiring of Specific Inhibitory Connections in Adult Neocortex

**DOI:** 10.1371/journal.pbio.1001798

**Published:** 2014-02-25

**Authors:** Dennis Kätzel, Gero Miesenböck

**Affiliations:** Centre for Neural Circuits and Behaviour, University of Oxford, Oxford, United Kingdom; University of California, Berkeley, United States of America

## Abstract

Although neocortical connectivity is remarkably stereotyped, the abundance of some wiring motifs varies greatly between cortical areas. To examine if regional wiring differences represent functional adaptations, we have used optogenetic raster stimulation to map the laminar distribution of GABAergic interneurons providing inhibition to pyramidal cells in layer 2/3 (L2/3) of adult mouse barrel cortex during sensory deprivation and recovery. Whisker trimming caused large, motif-specific changes in inhibitory synaptic connectivity: ascending inhibition from deep layers 4 and 5 was attenuated to 20%–45% of baseline, whereas inhibition from superficial layers remained stable (L2/3) or increased moderately (L1). The principal mechanism of deprivation-induced plasticity was motif-specific changes in inhibitory-to-excitatory connection probabilities; the strengths of extant connections were left unaltered. Whisker regrowth restored the original balance of inhibition from deep and superficial layers. Targeted, reversible modifications of specific inhibitory wiring motifs thus contribute to the adaptive remodeling of cortical circuits.

## Abstract 2

Optogenetic mapping of connectivity in the primary somatosensory cortex shows that mature cortical circuits adapt to sensory deprivation by selectively altering specific network motifs.

## Author Summary

Many natural and engineered networks contain recurring patterns of local connectivity. Although these so-called network motifs are thought to have functional significance, direct tests of the idea that network topology reflects function remain scarce. We have performed such a test in the area of mammalian neocortex that is devoted to the sensory representation of touch. To this end, we equipped inhibitory interneurons in the mouse with light-activated ion channels that allowed us to stimulate interneuron activity optically and record light-evoked inhibitory currents in their postsynaptic partners, thereby revealing maps of connectivity. We find that excitatory pyramidal cells in layer 2/3 of primary somatosensory cortex receive inhibition from GABAergic interneurons located in different cortical layers, with a characteristic balance of inhibitory connections from deep and superficial layers. Trimming the whiskers to remove sensory input in adult animals alters this balance—inhibitory connections from deep cortical layers are depleted, while inhibitory connections from superficial layers are augmented. These changes revert when the whiskers regrow, restoring the original balance between wiring motifs. This see-saw relationship between deep and superficial inhibition demonstrates that mature cortical circuits adapt to functional change by selectively altering specific network motifs.

## Introduction

Neocortex has a similar multilayered histology throughout [1],[2], and different cortical areas are able to adapt, depending on their inputs, to the normal function of other regions [3]. This versatility may reflect the existence of a “canonical” information-processing architecture, underpinned by stereotyped patterns of excitatory connectivity [2],[4].

The organization of inhibitory neocortical circuits also obeys principles of some generality. A recent survey of inhibitory-to-excitatory wiring patterns in primary motor (M1), somatosensory (S1), and visual cortex (V1) of the mouse uncovered 25 interlaminar connection motifs common to all three regions [5]. Whereas most of these motifs were found at comparable frequencies in all cortical areas, the abundance of four motifs varied widely: ascending inhibition from layer 5B (L5B) to L2/3 and L4, as well as from L6 to L5B, was prominent in V1 and S1, but not in M1; descending inhibition from L4 to L5A featured notably in S1. These motifs may therefore represent adaptations of a common blueprint to region-specific information-processing demands.

This interpretation raises several questions. Is the presence of a specific wiring motif linked to the particular type of input a cortical area receives? In other words, does the motif change when the type of input changes? If so, is plasticity limited to a critical developmental period, or does the capacity to adapt persist into adulthood? And how motif-specific is the change? Are variable wiring motifs inserted or removed on demand, akin to plug-in devices that add new functionalities, or are circuits reconfigured more broadly?

To answer these questions, we have analyzed and compared the laminar organization of inhibitory inputs to pyramidal neurons in L2/3 of adult mouse barrel cortex (S1) under physiological conditions, during sensory deprivation (whisker trimming), and after recovery (whisker regrowth). In agreement with recent observations in visual cortex [6]–[9], we found that sensory deprivation of adult barrel cortex induced changes in inhibitory circuits. Importantly, the nature of these changes was not only an overall reduction in cortical inhibition, as had been inferred from the decrease of inhibitory neuron spine and bouton numbers observed earlier. Instead, inhibitory connections from particular cortical layers underwent large, reversible, motif-specific, and sometimes antagonistic adjustments. Individual inhibitory network motifs are thus altered selectively and independently to adapt a cortical area to functional change.

## Results

**Figure 1:**
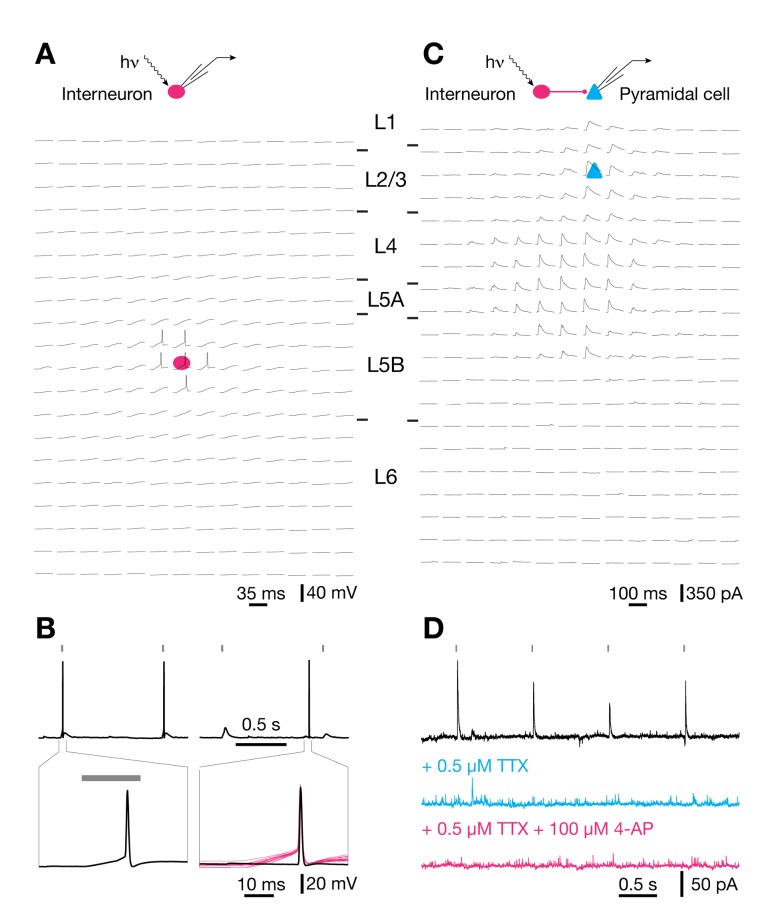
Optogenetic mapping of inhibitory connectivity. (A) Optical raster stimulation of GABAergic interneurons; electrophysiological recording from a L5B interneuron. Whole-cell current-clamp traces show light-evoked voltage responses as the focus of the stimulating beam is scanned across a grid of 14×20 locations. The somatic position of the recorded interneuron is indicated by a red ellipse; the grid spacing is 60 µm. Only perisomatic illumination evokes action potentials. (B) Waveforms of light-evoked (left) and spontaneous (right) action potentials at different timescales (top, bottom). Note that a slow depolarizing ramp to threshold after the onset of a light pulse (20 ms, gray bars) distinguishes light-evoked from spontaneous action potentials. The bottom right panel includes for comparison traces of all 15 light-evoked action potentials elicited during three repetitions of a raster scan (red traces, aligned to the time at which the rising action potential reached half-maximal amplitude). (C) Optical raster stimulation of GABAergic interneurons; electrophysiological recording from a L2/3 pyramidal cell. Whole-cell voltage-clamp traces show light-evoked IPSCs as the focus of the stimulating beam is scanned across a grid of 14×20 locations. The somatic position of the recorded pyramidal cell is indicated by a blue triangle; the grid spacing is 60 µm. (D) Waveforms of light-evoked IPSCs in a pyramidal cell voltage-clamped at 0 mV. IPSCs are blocked by bath application of TTX (blue) and fail to recover after additional application of 4-AP (red).

Experiments were performed on acute neocortical slices of a mouse knock-in line expressing the optogenetic actuator [10]–[12] channelrhodopsin-2 (ChR2, GenBank accession number AF461397; [13]–[15]) in inhibitory interneurons. ChR2 was present in ∼95% of GABAergic neurons, including virtually all cells positive for parvalbumin and somatostatin [5]. Blue illumination stimulated action potentials in >90% of all interneurons, with no detectable differences in optical responsiveness between subclasses (see [5] and below). Light pulses of 0.5–1.8 mW power and 20 ms duration evoked orthodromic spikes (which are distinguished by a slow depolarizing voltage ramp to threshold) when the focused stimulation beam was directed at the perisomatic region of interneurons (Figure 1A,B) but failed to elicit antidromic spikes (which show an abrupt onset because of active backpropagation to the somatic recording site) when the focus was swept across axonal compartments in the slice (Figure 1A,B). Consistent with the exclusively perisomatic generation of light-evoked action potentials [5], stimulus-locked inhibitory postsynaptic currents (IPSCs) in pyramidal cells (Figure 1C) vanished when voltage-gated sodium channels were blocked by tetrodotoxin (TTX) and failed to recover after additional blockade of repolarizing potassium channels by 4-aminopyridine (4-AP) (Figure 1D) [16]. Our mouse knock-in line thus offers a unique advantage over other expression systems [16]–[19] for mapping cell-to-cell connectivity in local circuits: Because illumination causes neither spiking in axons of passage nor local transmitter release from synaptic terminals, the somatic locations of connected partners can be identified unequivocally.

To determine the density and strength of inhibitory inputs from different cortical layers, pyramidal cells in L2/3 were voltage-clamped at 0 mV, while a focused laser beam scanned a pseudorandom pattern of 14×20 locations arranged in a 60-µm grid (Figure 1C). When a laser pulse elicited an action potential in a presynaptic partner, the voltage-clamp trace of the recorded L2/3 pyramidal cell showed an IPSC (Figure 1C,D) whose integrated charge was measured as an index of connection strength. The contribution of a cortical layer to the total amount of inhibition received by a target cell was quantified as the amount of inhibitory charge flowing from that layer into the target cell [5]. The absolute laminar charge flow is simply the product of the number of inhibitory input sources located in a given layer and the average integrated current (charge transfer) per IPSC. To obtain the normalized laminar charge flow, a convenient index of the relative laminar distribution of inhibitory inputs [5], the absolute charge flow was divided by the total inhibitory charge flow from all layers. Inputs were allocated to individual layers according to differences in cell density and shading in the bright-field image of the slice [5],[20]–[22].

**Figure 2:**
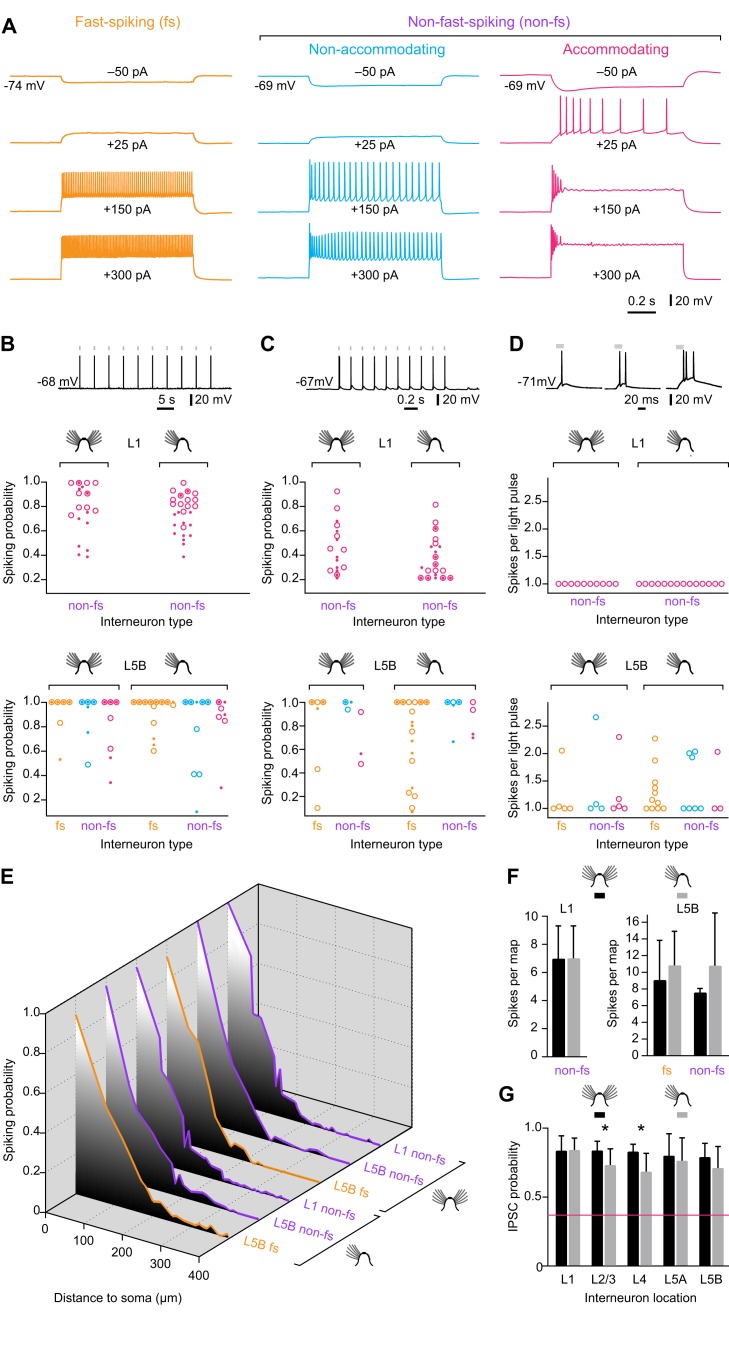
Light sensitivity of ChR2-expressing interneurons. (A) Classification of interneurons into fast-spiking (fs; left) and two types of non-fast-spiking (non-fs) cells, termed nonaccommodating or regular-spiking (center) and accommodating interneurons (right). Traces from top to bottom were recorded during 1-s current pulses of −50 pA, +25 pA, +150 pA, and +300 pA. (B, Top) Example of a spike train evoked by optical pulses (1.8 mW, 20 ms, gray bars) at a stimulation frequency of 0.2 Hz in a L5B interneuron. (Center and Bottom) Probability of evoking ≥1 spike per optical pulse during repeated optical stimulation (*n* = 3–5 trains of 10 light pulses each) of L1 (center) and L5B interneurons (bottom). Light pulses were presented at 0.2 Hz and carried optical powers of 0.5 mW (small solid circles) or 1.8 mW (large open circles); small solid within large open circles indicate identical spiking probabilities at both power levels. Interneuron responses were classified as fast-spiking (fs) or non-fast-spiking (non-fs) and recorded in control (left) or deprived conditions (right). No significant differences exist between any of these groups (*p*>0.05, *t* test). See Materials and Methods for criteria used to distinguish fs from non-fs cells. Yellow, fs interneurons; blue, nonaccommodating non-fs interneurons; red, accommodating non-fs interneurons. Only non-fs interneurons of the accommodating type were encountered in L1. (C) Same display as (B), but at 5 Hz stimulation frequency. No significant differences exist between any of the groups shown (*p*>0.05, *t* test). (D, Top) Examples of single spikes (left), spike doublets (center), and spike triplets (right) evoked by a single optical pulse in a train (1.8 mW, 20 ms, 0.2 Hz, gray bars). (Center and Bottom) Average number of spikes evoked per optical pulse in L1 (center) and L5B interneurons. Interneuron responses were classified as fast-spiking (fs) or non-fast-spiking (non-fs) and recorded in control (left) or deprived conditions (right). No significant differences exist between any of these groups (*p*>0.05, *t* test). See Materials and Methods for criteria used to distinguish fs from non-fs cells. Yellow, fs interneurons; blue, nonaccommodating non-fs interneurons; red, accommodating non-fs interneurons. (E) Spiking probabilities of L1 and L5B interneurons as functions of the distance of the stimulation spot from the soma, using the same pseudorandom 14×20-spot stimulation sequence as in mapping experiments (see Figure 1). The graphs represent data from 10 L1 interneurons, 5 non-fs L5B interneurons, and 5 fs L5B interneurons in control conditions, and from 13 L1 interneurons, 7 non-fs L5B interneurons, and 4 fs L5B interneurons in deprived conditions. (F) Same data as in (E), but displaying the average number of spikes evoked per 14×20-spot stimulation sequence for interneurons in L1 (left) and L5B (right). (G) Probability of evoking an IPSC during repeated optical stimulation (*n* = 8–10 trials) of the same presynaptic location in the indicated layers (*n* = 23 slices). Black columns, control condition; gray columns, deprived condition; error bars represent 1 SD; asterisks indicate significant differences (*p*<0.05, *t* test). The red line represents the most stringent criterion for detecting a synaptic input (≥3 IPSCs in eight trials; see Materials and Methods). In all cases, the reliability of transmission exceeds the criterion for detection by a wide margin.

With a lateral optical resolution of ∼60 µm FWHM (Figure 1A and Figure 2E) [5], a response reliability of >90% (Figure 2B) [5], a slice thickness of 310 µm, and a scatter coefficient of ∼10 mm^−1^ [23], we estimate that each light pulse activates ∼5–43 interneurons, depending on the cortical layer (Table S1) [24],[25]. The detection of an optically evoked IPSC signals the existence of a synaptic connection between at least one interneuron within the illuminated volume and the recorded pyramidal cell. The associated charge transfer is a compound measure reflecting the number of ChR2-expressing interneurons in the illuminated volume that are connected to the postsynaptic L2/3 pyramidal cell, the multiplicity of synapses between each presynaptic partner and the target cell, and the aggregate strength of these connections. Probing 14×20 locations in a raster scan (Figure 1C) generates an image of the laminar and columnar input distributions from ∼8,000 ChR2-expressing interneurons distributed over 3–4 barrel-related columns, which together occupy ∼0.25 mm^3^ of cortical tissue. This volume is at least an order of magnitude larger than what could realistically be sampled by two-photon random-access glutamate uncaging [26]. The mesoscopic scale of our type of analysis [5],[20]–[22],[27], however, comes at the expense of single-cell resolution. Our estimates of connection probability reflect the likelihood that an IPSC can be triggered by illuminating a particular volume of tissue, not a specific presynaptic cell. Similarly, our estimates of connection strength are based on the compound measure of integrated charge flow from an illuminated volume, not the physiological parameters of a single, identified synapse.

### Sensory Deprivation Alters the Laminar Organization of Inhibitory Circuits in a Motif-Specific Manner

Barrel cortex of adult mice was deprived of sensory input by trimming whisker rows A, B, D, and E every other day for 2–3 wk. Animals were aged 8–11 wk at the beginning of the manipulation and 10–14 wk at the time of analysis. To exclude potential confounds due to deprivation-induced changes in the optical excitability of ChR2-expressing interneurons, we compared the light responses of ChR2-positive cells in deprived barrel-related columns and nondeprived cortex. Our analysis concentrated on L5B, which exhibited the largest functional changes associated with deprivation (see below). We distinguished between fast-spiking and the two principal types of non-fast-spiking interneurons in layers 1 and 5, termed accommodating and nonaccommodating (or regular-spiking) cells (Figure 2A), and examined six measures of light sensitivity: the probability of evoking at least one action potential per pulse during optical pulse trains, which were delivered at 0.2 or 5 Hz, at power levels of 0.5 or 1.8 mW per pulse (Figure 2B,C); the average number of spikes evoked per 1.8 mW pulse during a 0.2 Hz train (Figure 2D); and the decay of light responsiveness as a function of the distance of the stimulating light beam from the soma (Figure 2E,F). Fast-spiking and non-fast-spiking interneurons behaved statistically indistinguishably in all six measures, which were all unperturbed by sensory deprivation. The passive membrane properties of the postsynaptic L2/3 pyramidal cells (Figure S1) were also unchanged in intact and deprived cortex, and the reliability of optically evoked transmission from all layers always exceeded the threshold for detecting a connection by a comfortable margin of safety (Figure 2G). Any experimentally induced changes in the structure of the inhibitory input maps we record therefore reflect changes in synaptic connectivity.

**Figure 3:**
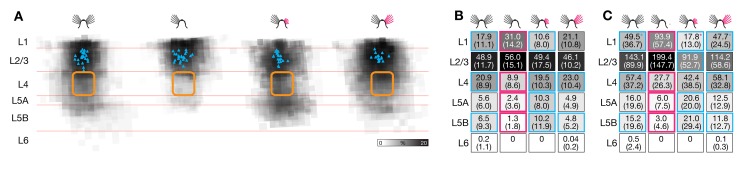
Sensory deprivation causes motif-specific changes in laminar inhibitory connectivity. (A) Maps of inhibitory inputs to L2/3 pyramidal neurons in columns representing intact (left, *n* = 23), trimmed (center left, *n* = 23), or previously deprived whiskers after regrowth for 1 mo (center right, *n* = 19) or 3 mo (right, *n* = 17). The maps are scaled to the size of a standard barrel (yellow outline) and overlaid to depict the distribution of inhibitory input sources. The intensity of gray shading at each location indicates the cumulative inhibitory charge transfer. This normalized index measures the frequency with which IPSCs are elicited from corresponding locations in different slices, weighted by the average charge transfer per IPSC. (B) Normalized inhibitory charge flow from the indicated source layers (rows) to L2/3 pyramidal neurons in columns representing intact (left, *n* = 23), trimmed (center left, *n* = 23), or previously deprived whiskers after regrowth for 1 mo (center right, *n* = 19) or 3 mo (right, *n* = 17). Values are represented numerically (±1 SD) and in normalized gray scale. Red outlines mark significant differences associated with whisker trimming (*p*<0.05; ANOVA); blue outlines indicate groups whose means differ from the whisker-trimmed state (Bonferroni-corrected *t* test). (C) Same display as (B), but illustrating absolute laminar inhibitory charge flow in pC (mean ± 1 SD). An additional significant difference exists in L1 between the 1-mo-regrowth condition (red asterisk) and columns representing intact and fully regrown whiskers (blue asterisks) (*p*<0.05; Bonferroni-corrected *t* test).

In barrel-related columns representing trimmed whisker rows A, B, D, and E, sensory deprivation was linked to a conspicuous loss of ascending inhibitory inputs to L2/3 pyramidal cells from deeper layers (Figure 3A and Figure S2). The normalized inhibitory charge flow from layers 4, 5A, and 5B decreased to 43%, 44%, and 19%, respectively, of control conditions (Figure 3B). Overall, the relative and absolute contributions of different cortical layers to the inhibitory charge flow of L2/3 pyramidal cells changed in a manner consistent with targeted adjustments of selected connections (Figure 3B,C). The most striking example of motif-specific plasticity was antagonistic changes occurring simultaneously to different inhibitory connections in the same barrel-related column: while inhibition from L5B weakened 5-fold in deprived columns, inhibition from L1 nearly doubled (Figure 3B,C).

After whiskers had been allowed to regrow for 3 mo, the inhibitory charge flow from all cortical layers returned to baseline values (Figure 3). Full whisker regrowth restored the original balance between L5B- and L1-derived inhibition, by strengthening the former and weakening the latter (Figure 3B,C). The antagonistic relationship between L1- and L5-derived inhibition held even during a transient stage of overcompensation when whisker regrowth was partial; at this stage, the aggregate strengths of inhibitory inputs from the two layers overshot their targets in opposite directions (Figure 3B,C).

### Sensory Deprivation Leaves the Horizontal Organization of Inhibitory Circuits Unperturbed

**Figure 4:**
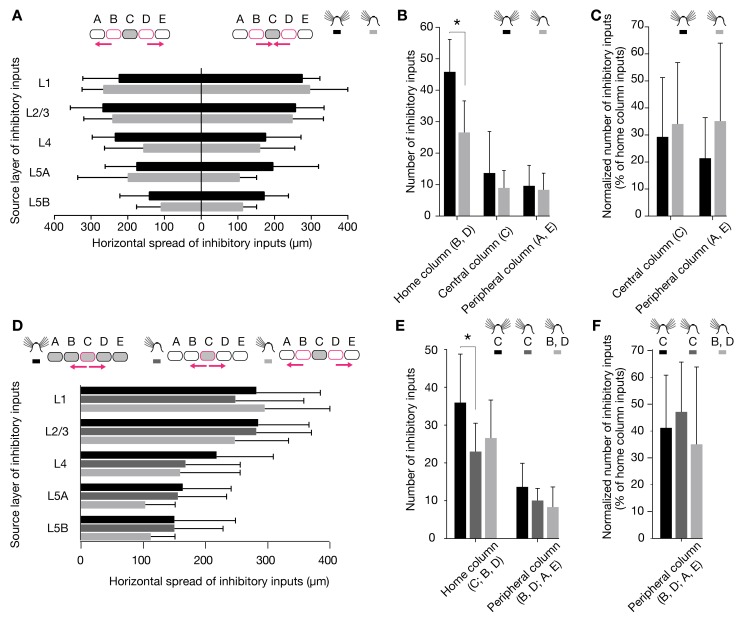
Horizontal distribution of inhibitory inputs among barrel-related columns. (A–C) Recordings were obtained from L2/3 pyramidal cells in barrel-related columns B (*n* = 4 cells in control and 2 cells in deprived conditions) or D (*n* = 8 cells in control and 15 cells in deprived conditions). (A) Average horizontal distance of the most distant inhibitory input from the center of the home column in the indicated layers. The left bar graph shows the horizontal spread of inhibitory inputs from the peripheral columns A or E, which represent trimmed whiskers; the right bar graph shows the horizontal spread of inhibitory inputs from the central column C, which represents an intact whisker. Black columns, control condition; gray columns, deprived condition; error bars represent 1 SD. No significant differences exist between any of the groups (*p*>0.05, *t* test). (B) Absolute numbers of connected locations in the home column; the peripheral columns A or E, which represent trimmed whiskers; and the central column C, which represents an intact whisker. Black columns, control condition; gray columns, deprived condition. Error bars represent 1 SD; the asterisk indicates a significant difference (*p*<0.05; *t* test). (C) Same display as (B), but depicting connected locations normalized to the number of locations in the home column. No significant differences exist between conditions (*p*>0.05, *t* tests). (D–F) Recordings were obtained from L2/3 pyramidal cells in barrel-related column C (*n* = 13 cells in control and 12 cells in deprived conditions). (D) Average horizontal distance of the most distant inhibitory input from the center of the home column in the indicated layers. The bar graph shows the horizontal spread of inhibitory inputs toward columns representing trimmed whiskers. Black columns, control condition; dark gray columns, spread from spared column C to deprived columns B or D; light gray columns, spread from deprived columns B or D to deprived columns A or E; error bars represent 1 SD. The data set depicted in light gray is reproduced from panel A. No significant differences exist between any of the groups (*p*>0.05, *t* test). (E) Absolute numbers of connected locations in the home column and an adjacent deprived column. Black columns, control conditions; dark gray columns, column representing an intact whisker in otherwise deprived conditions; light gray columns, column representing a trimmed whisker in deprived conditions. Error bars represent 1 SD; the asterisk indicates a significant difference (*p*<0.05; *t* test). (F) Same display as (E), but depicting connected locations normalized to the number of locations in the home column. No significant differences exist between any of the groups (*p*>0.05, *t* tests).

Sparing a single central whisker from deprivation can cause excitatory circuits to expand, so that signals from the spared whisker now activate surrounding deprived barrel-related columns [28],[29]. We examined if inhibitory circuits in columns representing intact whiskers similarly expanded into or retracted from deprived cortical areas. If this were the case, the inhibitory input distributions of whisker-related column rows B and D, which in our deprivation protocol neighbor the spared row C, would be expected to become asymmetric. No evidence for this type of territorial reorganization was found: neither the horizontal reach of inhibitory connections into deprived versus nondeprived barrel-related columns (Figure 4A), nor the number of inputs from these columns (Figure 4B,C), differed.

We also analyzed the horizontal inhibitory input distribution of L2/3 pyramidal neurons residing in the spared barrel-related columns of row C. Trimming the principal whiskers associated with adjacent barrel-related columns altered neither the horizontal spread (Figure 4D) nor the number of locations in deprived columns (Figure 4E,F) that gave rise to IPSCs in spared columns: the profile of horizontal inhibitory connections from deprived to spared columns was the same as that between deprived columns (Figure 4D). Our regime of sensory deprivation thus selectively altered the vertical (laminar) but not the horizontal (columnar) organization of inhibitory circuits.

**Figure 5:**
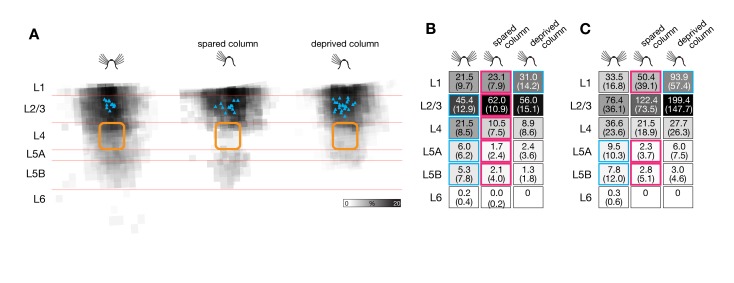
Sensory deprivation causes motif-specific changes in laminar inhibitory connectivity also in spared barrel-related columns. (A) Maps of inhibitory inputs to L2/3 pyramidal neurons in columns representing intact (left, *n* = 13, C column), spared (center, *n* = 12), or deprived whiskers (right, *n* = 23; same data as in Figure 3B). The maps are scaled to the size of a standard barrel (yellow outline) and overlaid to depict the distribution of inhibitory input sources. The intensity of gray shading at each location indicates the cumulative inhibitory charge transfer. This normalized index measures the frequency with which IPSCs are elicited from corresponding locations in different slices, weighted by the average charge transfer per IPSC. (B) Normalized laminar charge flow from the indicated source layers (rows) to L2/3 pyramidal neurons in barrel-related columns representing intact (left, *n* = 13, column C), spared (center, *n* = 12, column C), or deprived whiskers (right, *n* = 23, columns A, B, D, and E; same data as in Figure 3B). Values are represented numerically (±1 SD) and in normalized gray scale. Red outlines mark significant differences between the spared column in deprived cortex and either of the other two (blue) conditions (*p*<0.05, *t* test). (C) Same display as (B), but illustrating absolute laminar inhibitory charge flow in pC (mean ± 1 SD).

Remarkably, deprivation-induced changes in vertical inhibitory connectivity also affected the spared barrel-related columns of row C. As in deprived columns, the number of home column inputs decreased significantly (Figure 4E), but the detailed pattern of laminar reorganization differed subtly. Spared and deprived whisker columns suffered an equally sharp drop of inhibitory charge flow from the thalamorecipient layers 4 and 5A (Figure 5A). However, some inhibition from L5B was preserved in spared columns (Figure 5A), and the antagonistic increase of L1-derived inhibition was lacking (Figure 5B,C).

### Motif-Specific Changes in Connection Probability

Several mechanisms could generate these adaptations, singly or in combination. Elaboration or retraction of inhibitory terminals could alter the number of pyramidal cells contacted by one interneuron (a change in connection probability) or the number of synapses between one interneuron and one pyramidal cell (a change in connection strength). Differences in connection strength could also arise if synaptic release probability or quantal size were modulated. A formal, though remote, possibility is that the number of interneurons themselves might change during deprivation.

**Figure 6:**
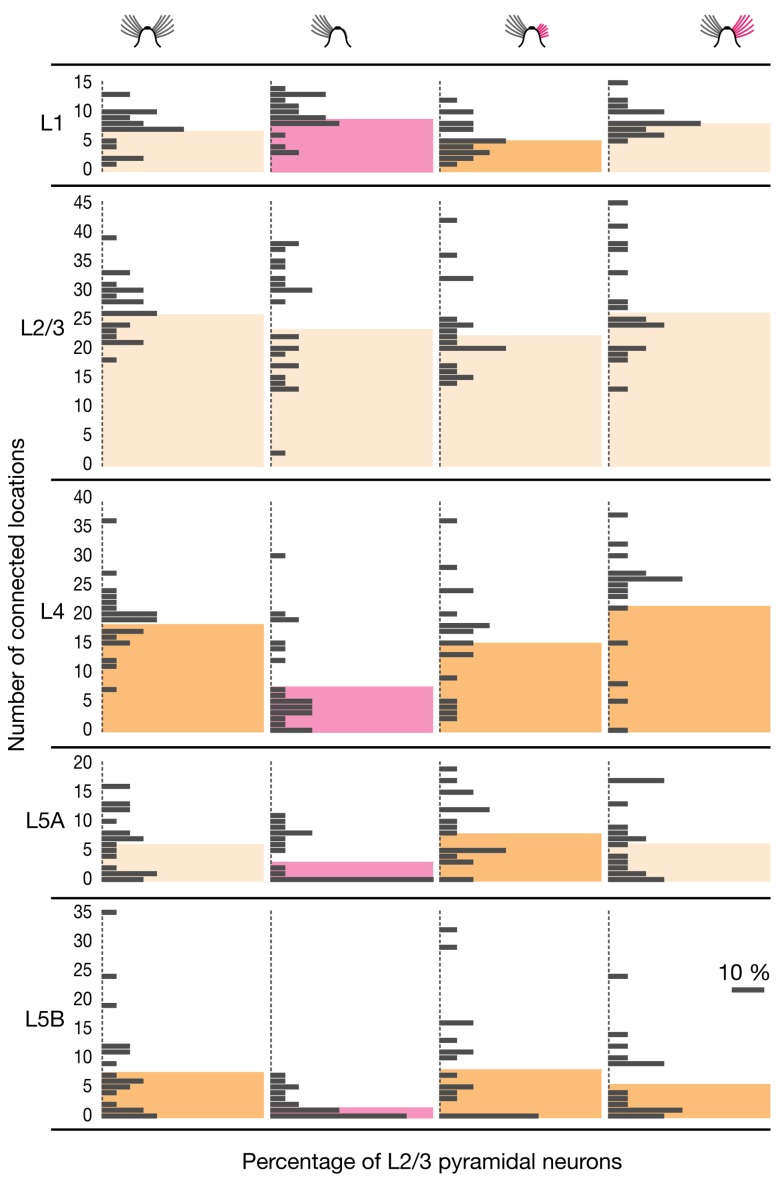
Sensory deprivation causes motif-specific changes in inhibitory input numbers. Absolute number of locations in the indicated source layers (rows) giving rise to IPSCs in L2/3 pyramidal neurons in barrel-related columns representing intact (left, *n* = 23), trimmed (center left, *n* = 23), and previously deprived whiskers after regrowth for 1 mo (center right, *n* = 19) and 3 mo (right, *n* = 17). Colored columns represent group averages. Red columns mark significant differences associated with whisker trimming (*p*<0.05; ANOVA); dark yellow columns indicate groups whose means differ from the whisker-trimmed state (Bonferroni-corrected *t* test). No significant differences exist between control and regrowth conditions (*p*>0.05; ANOVA).

In adult mice with intact or fully regrown whiskers, identically sized majorities of pyramidal cells in L2/3 were targeted by L5B interneurons (19/23, or 82.6%, of cells in control conditions; 14/17, or 82.4%, of cells after 3 mo of whisker regrowth). By contrast, in deprived barrel-related columns approximately one half (11/23) of L2/3 pyramidal cells lacked any detectable input from layer 5B (Figure 6). Where connections from L5B remained after deprivation, their numbers were severely, selectively, and reversibly depleted (Figure 6 and Figure S2). The average number of connected locations in L5B dipped from 8.0±9.9 in columns representing intact whiskers to 1.7±2.2 in deprived columns; input numbers returned to 8.5±9.5 and 5.9±6.6, respectively, during and after whisker regrowth (means ± 1 SD; *p* = 0.002; ANOVA). Most of the few surviving sources of inhibition attributed to L5B arose from stimulation sites that straddled the border to L5A (Figure 3A and Figure S2), raising the possibility that the depletion of inhibitory connections from L5B was virtually complete. In columns representing intact whiskers, in contrast, presynaptically connected interneurons populated the full depth of L5B (Figure 3A). IPSCs could be elicited from 13.4%±16.0% of L5B stimulation spots in control cortex and from 15.3%±17.3% in previously deprived columns after whisker regrowth, but only from 3.3%±4.4% of all L5B locations in deafferented columns (means ± 1 SD; *p* = 0.023; ANOVA).

Inhibition from layers 4 and 5A underwent qualitatively similar but less extensive changes. Absolute input numbers, as well as the fractions of connected locations, fell during sensory deprivation but rebounded fully when sensory input was restored (Figure 6). Connections from L1 followed a trend opposite to that of connections from deep layers. The average number of inhibitory inputs from L1 rose from 7.2±3.3 in control barrel cortex to 9.2±3.1 in deprived whisker columns, and returned to 5.5±3.1 and 8.5±3.5 during and after whisker regrowth, respectively (means ± 1 SD; *p*<0.001; ANOVA; Figure 6). These profound and antagonistic changes to four translaminar wiring motifs occurred against a backdrop of stable inhibitory-to-excitatory connectivity in the home layer. The number of inhibitory inputs from L2/3 fluctuated only marginally between a minimum of 23.2±9.7 after 1 mo of whisker regrowth and a maximum of 27.1±8.8 after 3 mo of regrowth (Figure 6).

**Figure 7:**
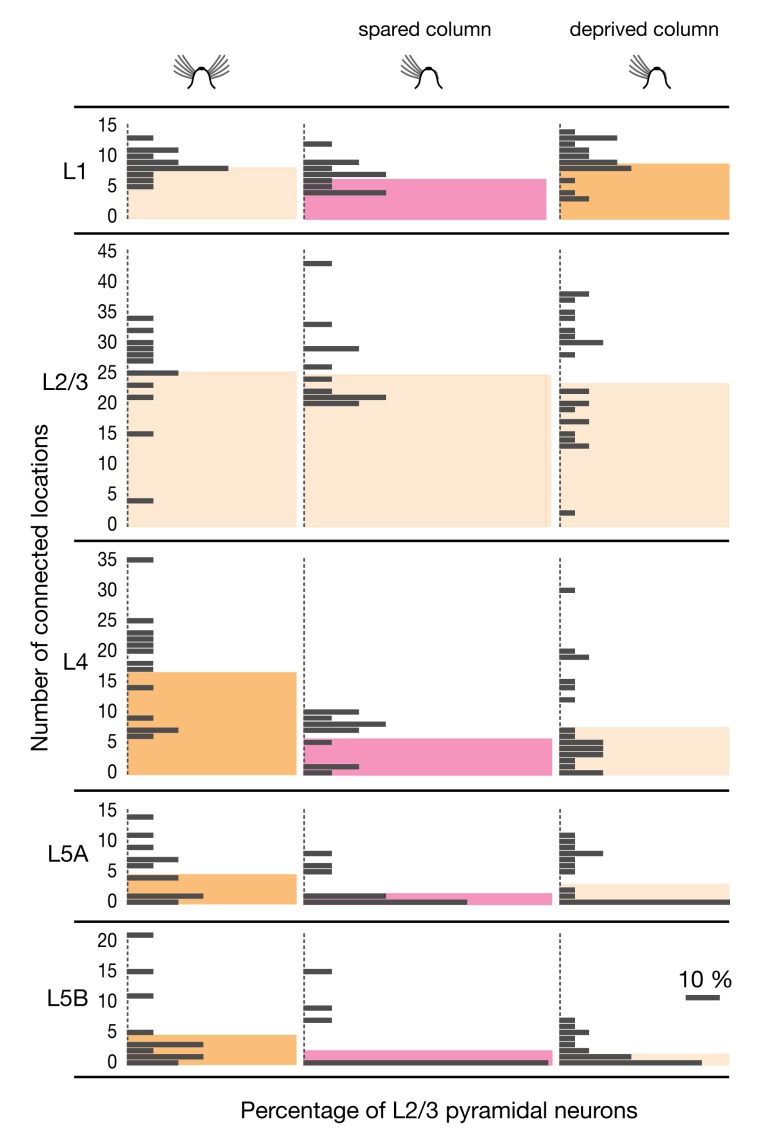
Sensory deprivation causes motif-specific changes in inhibitory input numbers also in spared barrel-related columns. Absolute number of locations in the indicated source layers (rows) giving rise to IPSCs in L2/3 pyramidal neurons in barrel-related columns representing intact (left, *n* = 13, column C), spared (spared, *n* = 12, column C), or deprived whiskers (right, *n* = 23, columns A, B, D, and E; same data as in Figure 6, center left). Colored columns represent group averages. Red columns mark significant differences between the spared barrel-related column in deprived cortex and the comparison groups indicated in dark yellow (*p*<0.05, *t* test).

In contrast to the antagonistic relationship between inhibitory inputs from superficial and deep cortical layers in deprived barrel-related columns (Figure 6 and Figure 7), translaminar inhibitory connections in the spared whisker-related row C of deprived cortex became uniformly sparse. In deep cortical layers, the number of connected locations dropped significantly: from 17.2±8.5 to 6.2±3.6 in L4, from 5.0±4.5 to 1.8±2.8 in L5A, and from 5.1±6.5 to 2.6±5.0 (means ± 1 SD; *p*<0.05; *t* test; Figure 7). The percentages of stimulation spots from which IPSCs could be elicited decreased from 34.7% to 11.4% in L4, from 23.9% to 8.2% in L5A, and from 10.2% to 4.8% in L5B (*p*<0.05, *t* test). Even in L1 the number of connected locations fell slightly (Figure 7), in keeping with the lack of a deprivation-induced surge in inhibitory charge flow from that layer (Figure 5B,C).

**Figure 8:**
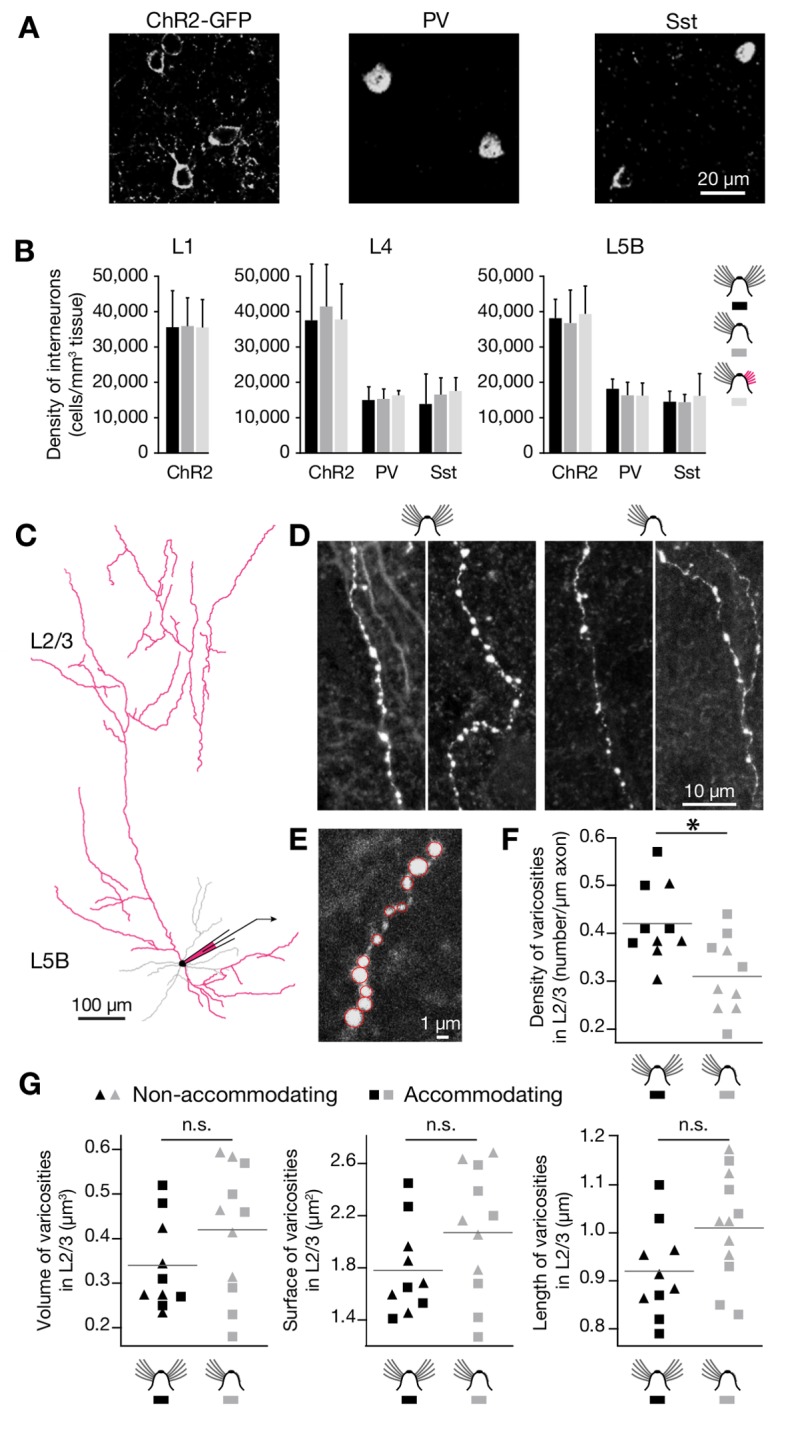
Sensory deprivation reduces the density of presynaptic boutons formed by L5B interneurons in L2/3. (A) Slices of barrel cortex were immunolabeled with antibodies against GFP (left), parvalbumin (PV, center), and somatostatin (Sst, right). The examples show raw confocal images of L5B after staining with fluorescently labeled secondary antibodies. (B) Densities of cells expressing ChR2-GFP, PV, and Sst in L1 (left), L4 (center), and L5B (right) of barrel-related columns representing intact, trimmed, or previously deprived whiskers after regrowth for 1 mo (mean ± 1 SD; *n* = 5–16 confocal stacks from 2 mice per condition). No significant differences exist between any of these conditions in any of the layers (*p*>0.05; ANOVA). (C) Experimental configuration for axonal morphometry. Interneurons located in L5B were filled with neurobiotin to visualize their axonal arbors in L2/3 (reconstructed in red in the example shown). (D) Examples of axon segments in L2/3 deriving from four different neurobiotin-filled interneurons located in L5B. The linear density of presynaptic varicosities differs between control (left panels) and deprived (right panels) conditions. (E) A higher magnification view shows that individual presynaptic varicosities (red circles) can be distinguished unambiguously. (F) Average linear density of presynaptic varicosities along L5B interneuron-derived axon segments in L2/3 (*n* = 10 segments each in control and deprived conditions; total reconstructed axon length 7,071 and 3,204 µm, respectively). Black symbols, control condition; gray symbols, deprived condition; triangles, nonaccommodating non-fs interneurons; squares, accommodating non-fs interneurons. The asterisk indicates a significant difference (*p*<0.05, *t* test). (G) Average volume (left), surface area (middle), and diameter (right) of presynaptic varicosities along L5B interneuron-derived axon segments in L2/3 (*n* = 10 segments and 600 boutons each in control and deprived conditions). Black symbols, control condition; gray symbols, deprived condition; triangles, nonaccommodating non-fs interneurons; squares, accommodating non-fs interneurons. No significant differences exist between the groups (*p*>0.05, *t* tests).

Our experimental manipulations altered neither the optical excitability of ChR2-expressing interneurons (Figure 2B–G) nor the reliability of optically evoked synaptic transmission (Figure 2H). The rearranged inhibitory input maps of L2/3 pyramidal cells in deprived cortex must therefore reflect changes in the number or subclass distribution of presynaptic interneurons, or changes in connection probabilities between these neurons and their postsynaptic targets. Immunohistochemistry ruled out the first mechanism: neither the densities of ChR2-expressing interneurons in the plastic layers 1, 4, and 5, nor the distributions of the major subpopulations of parvalbumin- and somatostatin-positive cells, changed (Figure 8A,B). The measured variations in the number and locations of sites where IPSCs could be stimulated are thus indicative of changes in connection probabilities.

To search for structural correlates of these functional changes, we analyzed the wiring motif undergoing the largest deprivation-induced change: ascending inhibition from L5B (Figure 3 and Figure 6). Forty-nine interneurons in L5B were filled with neurobiotin; of these, 11 cells showed high-contrast axonal staining in upper cortical layers (Figure 8C,D). All of these 11 cells were non-fast-spiking interneurons of the accommodating (*n* = 6) or regular-spiking (nonaccommodating) type (*n* = 5), consistent with the notion that non-fast-spiking Martinotti cells are the principal conduits of L5-to-L2/3 inhibition [30],[31]. The neurobiotin-filled axons of L5B interneurons extending into L2/3 carried fewer varicosities per unit length in deprived cortex than did their counterparts in control conditions, as would be expected if presynaptic terminals were eliminated following sensory deprivation (Figure 8D–F). There were no statistically significant differences between the volumes, surface areas, and maximal diameters of varicosities in deprived and control conditions; qualitatively, varicosities in deprived cortex even appeared somewhat larger than in intact cortex (Figure 8G).

The fractional loss of inhibitory varicosities after whisker trimming was, however, smaller than the fractional reduction in inhibitory charge flow from L5B (compare Figure 3B, Figure 6, and Figure 8F). This apparent mismatch may be accounted for in several ways. First, it is conceivable that varicosities remain visible after deprivation but the associated synapses have fallen silent. Second, connections with pyramidal cells may represent only a fraction of all synapses formed by L5B interneurons in L2/3. If only synapses with pyramidal cells are eliminated after deprivation, an aggregate varicosity count will underestimate the magnitude of this change. Third, whole axonal branches might be retracted. Although axonal pruning is considered an unlikely mechanism of lesion-induced inhibitory plasticity in adult visual cortex [7], it remains a formal possibility in barrel cortex.

### Motif-Specific Changes in Connection Strength

**Figure 9:**
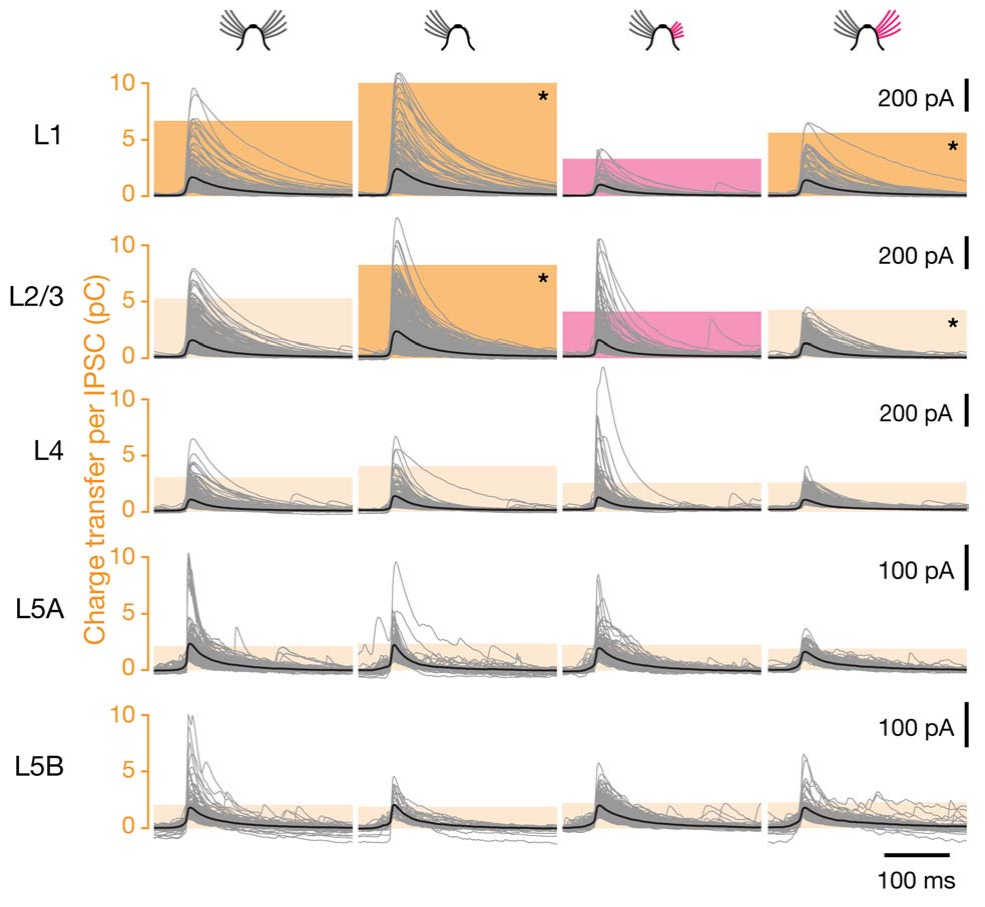
Sensory deprivation causes motif-specific changes in inhibitory connection strength. Voltage-clamp recordings at a holding potential of 0 mV from L2/3 pyramidal neurons in barrel-related columns representing intact (left, *n* = 23), trimmed (center left, *n* = 23), or previously deprived whiskers after regrowth for 1 mo (center right, *n* = 19) or 3 mo (right, *n* = 17). IPSCs were evoked by optical stimulation of interneurons in the indicated cortical layers; traces of all individual IPSCs (gray) were aligned to the time at which the rising IPSC reached half-maximal amplitude. Bold black traces indicate group averages. Colored columns represent the mean integrated current (charge transfer) per IPSC. Red columns mark significant differences associated with partial whisker regrowth (*p*<0.05; ANOVA); dark yellow columns indicate groups whose means differ from the state of partial whisker regrowth (Bonferroni-corrected *t* test). Asterisks mark pairwise differences remaining after whisker regrowth for 3 mo (*p*<0.05; ANOVA followed by Bonferroni-corrected *t* test). See also Figure S3 and Figure S4.

**Figure 10:**
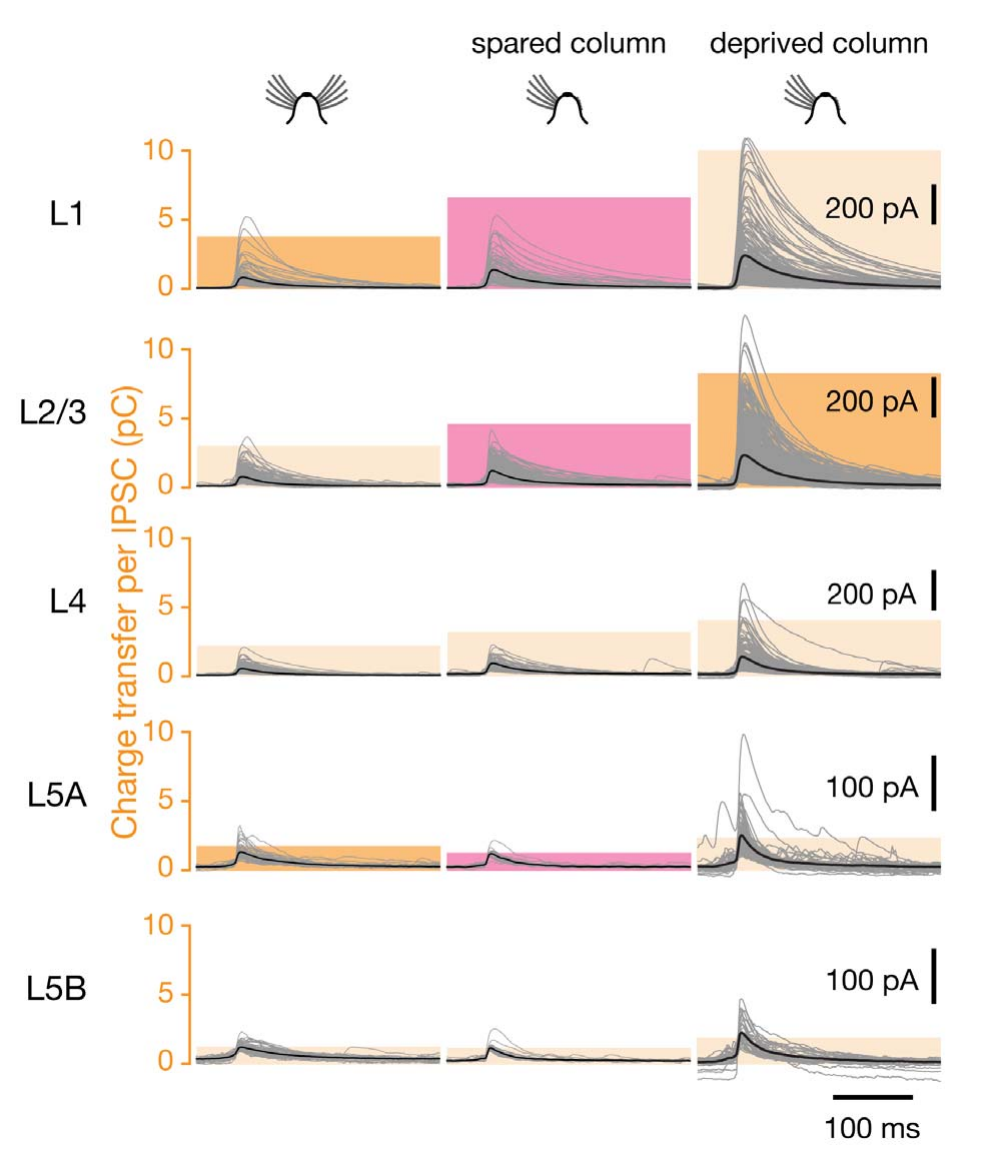
Sensory deprivation causes motif-specific changes in inhibitory connection strength also in spared barrel-related columns. Voltage-clamp recordings at a holding potential of 0 mV from L2/3 pyramidal neurons in barrel-related columns representing intact (left, *n* = 13, column C), spared (center, *n* = 12), or deprived whiskers (right, *n* = 23; same data as in Figure 9, center left panels). IPSCs were evoked by optical stimulation of interneurons in the indicated cortical layers; traces of all individual IPSCs (gray) were aligned to the time at which the rising IPSC reached half-maximal amplitude. Bold black traces indicate group averages. Colored columns represent the mean integrated current (charge transfer) per IPSC. Red columns mark significant differences between the spared barrel-related column in deprived cortex and the comparison groups indicated in dark yellow (*p*<0.05, *t* test).

Interneurons are thought to innervate each of their postsynaptic targets via multiple boutons (typically ∼15) [30],[32],[33]. Whisker deprivation might cause some of these boutons to be eliminated at random. The loss of a measurable connection would then simply result from the stochastic depletion of all synapses between two locations. If boutons were indeed silenced or pruned in this shotgun manner, surviving connections would be expected to suffer partial bouton losses and, therefore, be weaker than those in control conditions. Surprisingly, this was not the case: sensory deprivation left the strengths of extant connections from deep layers, measured as the mean integrated current per IPSC, virtually unchanged (Figure 9 and Figure 10). Importantly, the coefficients of variation of the individual IPSC amplitudes also remained unchanged (Figure S3). It is therefore implausible that different subsets of inhibitory synapses underwent large but opposite adjustments that canceled one another in the average.

Of course, optical stimulus-locked IPSCs may represent compound events if multiple presynaptically connected interneurons are activated simultaneously. Because the mean integrated current depends on the number and the individual strengths of all contributing synapses, it cannot be equated with the strength of a monosynaptic connection. In several instances, however, the two variables of synapse number and average synapse strength could be disambiguated by considering changes in charge transfer in the context of simultaneously occurring changes in the number of connected locations. For example, sensory deprivation greatly reduced the number of connections from layers 4 and 5 but left the charge transfer per remaining connection unchanged. If it is reasonable to assume, in light of this general trend toward synapse elimination, that persisting connections will be made through a constant or smaller rather than a larger number of synapses, then the average strength of these synapses must remain level or increase after deprivation. Our morphometric finding that L5B-derived axonal varicosities in deafferented whisker columns retained their pre-deprivation size or even expanded slightly (Figure 8G) reinforces this conclusion, as bouton size and synaptic strength tend to be tightly correlated [34].

An analogous argument applies to L1 of the spared whisker-related row C. The charge transfer per IPSC in these barrel-related columns increased significantly after whisker trimming, while the number of connected locations fell marginally (Figure 7 and Figure 10). This constellation of changes indicates that the average L1-derived synapse gained in strength. In L1 of deprived columns, in contrast, both the average charge transfer per IPSC and the number of connected locations rose insignificantly. The statistically significant increase in laminar charge flow from L1 after whisker trimming (Figure 3) thus remains an unresolved consequence of combined increases in monosynaptic connection probability and monosynaptic connection strength.

A history of deprivation had profound aftereffects on the strengths of inhibitory inputs originating in superficial layers 1 and 2/3. The inhibitory charge flow per IPSC from these layers increased marginally upon sensory deprivation, echoing similar changes during the critical period [35], but plummeted during subsequent whisker regrowth (Figure 9 and Figure S4). In contrast to the rapid and complete resurgence of inhibitory input numbers upon sensory restoration (Figure 6), the recovery of connection strengths was delayed and partial, even after 3 mo of whisker regrowth (Figure 9 and Figure S4). Although the causes and significance of this hysteretic effect are currently unknown, the phenomenon provides clear further evidence that sensory plasticity operates by tuning probabilities and strengths of synaptic connections independently of each other.

## Discussion

The adaptations documented here lay bare four remarkable features of experience-dependent plasticity of inhibitory connections. First, extensive changes take place in adult neocortex, long after the critical period for refining neuronal connections has closed [29]. Second, different wiring motifs are altered selectively and independently of one another. The most compelling illustration of this principle is the see-saw relationship between L1- and L5B-derived inhibition in deprived cortex: upon whisker trimming and regrowth, connections to a common postsynaptic target, the L2/3 pyramidal neuron, undergo simultaneous but opposite functional changes (Figure 3). Third, adjustments of inhibitory connection probabilities are fully reversible upon sensory restoration, even in cases where entire connections appear to have been lost during deprivation (Figure 3 and Figure 6). The removal of a measurable connection may thus not entail the physical retraction of axonal and/or dendritic branches, as is the case in critical period plasticity [36],[37], but rather the shutdown of transmission between synaptic partners that remain in close apposition [7],[38]. Fourth, probability and strength of a connection are independent dimensions for functional adjustment (Figure 6, Figure 7, Figure 9, and Figure 10).

Although the experimental settings and analytical approaches differ, it is instructive to compare our present findings with those of previous reports of experience-dependent inhibitory plasticity in adult neocortex [6]–[9],[39]. With one exception [39], all of these studies have examined the dynamics of structural changes in visual cortex following retinal lesions or monocular deprivation. Chronic imaging of the dendritic and axonal arbors or fluorescently tagged synapses of L1 and L2/3 interneurons revealed a seemingly general, rapid, and lasting loss of dendritic spines or branch tips [6],[7], axon terminals [7], and gephyrin-labeled postsynaptic puncta [8],[9] after deprivation. These morphological changes were taken to indicate a broad adaptive downscaling of inhibition. Our direct measurements of activity-induced functional changes in inhibitory-to-excitatory connections across the entire depth of somatosensory cortex paint a more differentiated picture. Although we do find a general decrease in the number of cortical locations providing inhibitory inputs to L2/3 pyramidal neurons (from 67.7±17.0 in control columns to 46.4±19.4 in whisker-deprived columns; means ± 1 SD; *p*<0.001, *t* test; Figure 3B), the scale and specificity of cortical remodelling become apparent only when individual wiring motifs are disentangled and analyzed separately (Figure 3).

From hindsight, hints of motif-specific plasticity are already evident in some earlier studies of excitatory [21],[40],[41] and even inhibitory cortical connections. For example, although the net elimination rate of boutons originating from L2/3 interneurons was found to increase after visual deprivation, inhibitory synapses onto L2/3 pyramidal cells—as opposed to those targeting dendrites of layer 5 pyramidal cells—appeared exempt from elimination [6],[7]. These results are consistent with the stability of home layer-derived inhibition in our hands (Figure 3, Figure 6, and Figure 9). Another example is the distinct behavior of different populations of inhibitory axons in superficial layers of barrel cortex. After whisker plucking, some axons in deprived barrel-related columns sprout, while others in the same column suffer bouton losses [39]. In light of our observations it is likely that the sprouting axons derive from L1 interneurons, while axons suffering bouton losses originate from interneurons in deep layers that target the apical dendrites of L2/3 pyramidal cells (Figure 3B).

**Figure 11:**
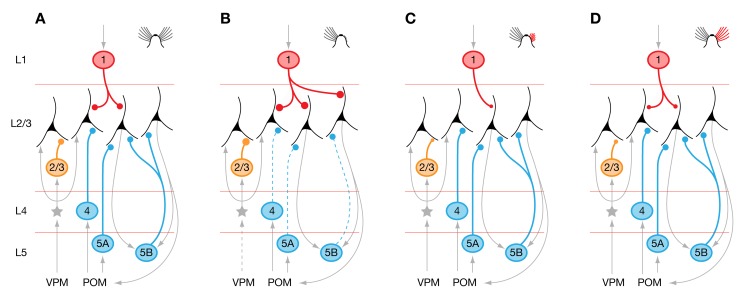
Plasticity of inhibitory wiring motifs in a functional context. (A) L2/3 pyramidal cells in barrel cortex of mice with intact whiskers receive feed-forward (yellow), feedback (blue), and top-down inhibition (red). Interneurons providing these different forms of inhibition are located in different cortical layers, where they are driven, respectively, by thalamocortical afferents and cortical excitatory neurons (L4 and L2/3 interneurons, yellow); by intracortical or cortico-thalamo-cortical loops (L4 septal and L5B interneurons, blue); and by interareal excitatory connections terminating in L1 (red). (B) Whisker trimming leads to motif-specific changes in connection probabilities: existing inhibitory connections from L4 and L5B are eliminated, whereas new inhibitory connections from L1 are formed. (C) Partial whisker regrowth leads to overcompensation of deprivation-induced changes in connection probabilities and a large reduction in the strength of inhibitory connections from L1 and L2/3. (D) Full whisker regrowth restores connection probabilities to baseline. The strength of inhibitory connections from L1 and L2/3 recovers only partially.

The adaptive changes displayed by different inhibitory circuit motifs offer some clues to the possible roles of these motifs in normal cortical function. Interneurons in L2/3 are thought to be driven by L2/3 excitatory neurons and the thalamocortical input layers 4 and 5A [27],[35]. The amount of feed-forward inhibition these neurons impose on L2/3 pyramidal cells is thus expected to scale with the excitatory drive to the column: the smaller the intensity of sensory stimulation, the smaller the inhibitory counterforce generated by the local feed-forward circuit (Figure 11) [35]. This autoregulatory feature may obviate the need for plasticity of L2/3-derived inhibitory connections and explain their relative stability in the face of our experimental perturbations (Figure 3 and Figure 6).

The inhibitory L5B-to-L2/3 motif, in contrast, is all but deleted from deprived whisker columns (Figure 3 and Figure 6). This striking adaptation suggests that the inhibition imposed by L5B interneurons on L2/3 pyramidal cells would prove excessive if the connection were left in place unaltered. The loss of significant excitatory drive from nonprincipal whiskers may explain why the same adaptation occurs, albeit to a lesser extent, also in spared columns that border deprived cortical tissue (Figure 5 and Figure 7). Most L5B interneurons—including Martinotti cells, the putative mediators of ascending translaminar inhibition [30],[31]—lack an autoregulatory mechanism that couples their activity directly to the intensity of sensory stimulation [42]. Instead, L5B interneurons are likely to integrate signals from L2/3 networks spanning more than one cortical column [43] and relay this information back to L2/3 in the form of recurrent inhibition [31]. This type of supralinear feedback inhibition [31],[44], triggered by spontaneous activity or sensory input to nearby columns representing intact whiskers, would be expected to extinguish any residual sensory signals reaching columns whose associated principal whiskers have been trimmed. Unplugging the inhibitory feedback connection may be necessary to enable these columns to process feeble thalamic input (Figure 11).

Qualitatively similar considerations may apply to inhibition originating in layers 4 and 5A. L5A and septal regions of L4 form part of a cortico-thalamo-cortical loop involving the posterior medial nucleus (POM) [45]–[47]. Residual sensory input, relayed via POM to inhibitory neurons in layers 4 (septum) and 5A, might cause excessive inhibition in deprived barrel-related columns. The adaptations we observe may help to rebalance excitation and inhibition (Figure 11).

Inhibitory interneurons in L1, in contrast, are likely mediators of top-down control of cortical areas by hierarchically higher regions [48]–[50]. The increase in L1-derived inhibition after whisker trimming (Figure 3, Figure 6, and Figure 9) could represent an adaptation to the emergence of aberrant spontaneous activity in columns that are no longer properly modulated by sensory signals. Enhanced top-down inhibition may be needed to suppress this activity or prevent it from spilling into other sensory or sensorimotor structures (Figure 11) [51]. Even when surrounded by deprived cortex, columns with spared principal whiskers are exempt from this adaptation (Figure 5 and Figure 7), presumably because their main sensory afferent remains intact.

Plasticity that is wiring motif–, cell type– [40],[41], or input-specific [21] illustrates the modularity of neural organization more directly than did earlier, statistical analyses of neuronal connectivity [5],[52]–[54]. Scrutiny of the connection matrices of compact nervous systems [52], cortical areas [53], or local microcircuits [54] revealed that some patterns of interconnectivity are vastly overrepresented in comparison to their expected frequencies in random graphs. These so-called network motifs [52] have been proposed to represent functional modules, which are cascaded differently in circuits with different information-processing capabilities. Our demonstration that individual inhibitory motifs are selectively altered to accommodate new function lends credence to this proposal.

## Materials and Methods

### Ethics Statement

All procedures complied with the UK Animals (Scientific Procedures) Act 1986.

### Experimental Animals and Whisker Deprivation

Experimental animals were knock-in mice homozygous for *R26::CAG-lox-STOP-lox-ChR2-EGFP* responder and *Gad2::CreER^T2^* driver transgenes at both targeted loci [5]. Following tamoxifen induction of Cre recombinase activity, these animals express channelrhodopsin-2 (ChR2; GenBank accession number AF461397, [13],[14]) comprehensively in all main subclasses of GABAergic interneurons defined cytochemically [5]. Mice were maintained in top-open cages on a 12 h light/dark cycle and fed a custom diet based on Teklad 2018, but with vitamin A levels elevated to 100 IU/g (Harlan Laboratories). At 8–10 wk of age, whiskers in rows A, B, D, and E on the right side of the snout were trimmed every other day for 2–3 wk. Whisker trimming was performed under transient anesthesia induced by subcutaneous (s.c.) injection of ∼20 µl of a 3∶5 mixture of ketamine (100 mg/ml; Fort Dodge) and medetomidin (1 mg/ml; Pfizer) and reversed by s.c. injection of 15–20 µl atipamezole (5 mg/ml; Pfizer). Control animals underwent the same anesthetic regimen as did whisker-trimmed animals.

Slices of acutely deprived somatosensory cortex were harvested no later than 36 h after the last whisker trimming session. To examine the effects of recovery from deprivation, whiskers were allowed to regrow for 4–5 wk (1-mo regrowth) or 12–14 wk (3-mo regrowth). Starting at 6–8 d before slices were cut, mice were injected intraperitoneally (i.p.) on 5 consecutive days with 0.3–0.5 mg 4-OH-tamoxifen (Sigma-Aldrich), which was dissolved in sterile sunflower oil at 5 mg/ml.

### Electrophysiology and Optical Stimulation

Experiments were performed on mice 2–4 d after the last 4-OH-tamoxifen injection. Animals were anesthetized by i.p. injection of 150 µl of a 3∶5 mixture of ketamine (100 mg/ml; Fort Dodge) and medetomidin (1 mg/ml; Pfizer) and perfused cardially with ice-cold solution containing (in mM): 2.5 KCl, 1.25 NaH_2_PO_4_, 25 NaHCO_3_, 10 glucose, 240 sucrose, 0.5 CaCl_2_, 7 MgCl_2_, pH 7.4, 320 mOsm. The brain was recovered into perfusion solution, and 310-µm slices of the left primary somatosensory cortex (S1) were cut on a Leica VT1200S vibratome. The cutting plane was oriented across whisker barrel rows, maintaining angles of 45° with the midline and nearly 90° with the cortical surface [21],[55]. Slices were incubated in the dark for 1 h at 34°C and subsequently maintained, shielded from light, at 25°C in modified artificial cerebrospinal fluid (aCSF) containing (in mM): 125 NaCl, 2.5 KCl, 1.25 NaH_2_PO_4_, 25 NaHCO_3_, 25 glucose, 1.25 CaCl_2_, 2 MgCl_2_, pH 7.4, 315 mOsm. Recordings were performed at room temperature in aCSF containing (in mM): 125 NaCl, 3.5 KCl, 1.25 NaH_2_PO_4_, 25 NaHCO_3_, 25 glucose, 1.25 CaCl_2_, 1 MgCl_2_, pH 7.4, 310 mOsm. All extracellular solutions were bubbled with 95% O_2_/5% CO_2_.

Whole-cell recordings were obtained from cells in barrel-related columns corresponding to whisker rows A through E. Patch pipettes had tip resistances of 4–6 MΩ. The internal solution for voltage-clamp recordings from pyramidal cells contained (in mM): 110 CsOH, 110 gluconic acid, 0.2 EGTA, 30 Hepes, 2 MgATP, 0.3 Na_2_GTP, 4 NaCl, 5 QX-314-Br, 0.2% neurobiotin, pH 7.25, 274 mOsm. The internal solution for current-clamp recordings from interneurons contained (in mM): 120 K-gluconate, 10 KCl, 10 Hepes, 4 MgATP, 0.3 Na_2_GTP, 10 phosphocreatine, 0.2% neurobiotin. Signals were amplified and low-pass-filtered at 2 kHz by a Multiclamp 700a amplifier (Molecular Devices) and digitized at 5–10 kHz (Digidata 1440, Molecular Devices).

Three criteria were used to distinguish fast-spiking (fs) from non-fast-spiking (non-fs) interneurons. Fs neurons (i) attained firing rates >90 Hz during a 1,000-ms depolarizing current step, (ii) exhibited a ratio of >0.7 of the average interspike interval (ISI) at the beginning and end of the depolarizing current step (averages of 3 ISIs each), and (iii) displayed a spike width of ≤1 ms at half-maximal amplitude. Cells that met all three criteria were classified as fs and cells that failed all three criteria as non-fs.

Optical stimulation experiments were performed on a Zeiss Axioskop 2FS microscope. A 40×, 0.8 NA water immersion objective with DIC optics was used for electrode placement and a 10×, 0.3 NA water immersion objective, without DIC optics, for optical stimulation. The output of a continuous-wave solid-state laser with a maximum power of 325 mW at 473 nm (LRS-473-AH-300-10, Laserglow) was digitally switched and intensity-modulated by an acousto-optic deflector (IntraAction model ASN-802832 with ME-802 driver), positioned by a pair of galvanometric mirrors (GSI Lumonics VM500 with MiniSAX servo controllers), and merged with the epi-illumination path of the microscope via custom-built optics. Light pulses carried 0.5–1.8 mW of optical power at the exit pupil of the objective. To generate maps of inhibitory inputs, a virtual instrument written in LabVIEW 8.5 delivered focused stimulation light pulses (spot size 3–5 µm, 20 ms duration) at intervals of 680 ms to 60-µm grids encompassing 14×20 locations in pseudorandomized order.

### Immunohistochemistry

Animals (*n* = 2 in each condition) were perfused with phosphate-buffered saline (PBS, pH 7.4) containing 4% (w/v) paraformaldehyde (PFA) and 0.2% (v/v) picric acid under general ketamine-medetomidine anesthesia. The brain was removed, incubated for 24 h in perfusion solution, and infiltrated with 30% (w/v) sucrose in PBS for at least 24 h. Coronal sections of 50 µm were cut on a Leica SM 2000R sliding microtome. The sections were rinsed three times in Tris-buffered saline (TBS, Sigma), three times in TBS containing 3% (w/v) Triton X-100 (TBS-T), and once for 1 h in TBS-T containing 20% (v/v) horse serum (Vector Labs) and then incubated for 48 h at 4°C in TBS-T containing 1% horse serum and combinations of the following primary antibodies: anti-GFP (chicken, 1∶500, AbCam), anti-parvalbumin (mouse, 1∶2,000, Swant), or anti-somatostatin (rabbit, 1∶500, Millipore). The sections were rinsed 4 times in TBS and stained in TBS-T containing 1% horse serum and Alexa488- and Alexa546-labeled secondary antibodies (Invitrogen). After four rinses in TBS, the slices were mounted in VectaShield (Vector Labs) and imaged on a Leica TCS SP5 confocal microscope.

### Axonal Morphometry

To estimate the density of presynaptic varicosities along axon segments, the neurobiotin concentration in the internal solution for current-clamp recordings was raised to 1%. Patch configurations were converted to outside-out after neurobiotin infusion to allow the plasma membrane to reseal. Slices were incubated in modified aCSF for 1 h and then overnight in PBS containing 4% (w/v) PFA and 0.2% (v/v) picric acid. The slices were rinsed in TBS and stained in TBS-T containing 1% (v/v) horse serum, 4 µg/ml Alexa546-labeled streptavidin (Invitrogen), and 0.0001% DAPI (Sigma) for 12–24 h. After four rinses in TBS, the slices were mounted in VectaShield (Vector Labs) and imaged on a Leica TCS SP5 confocal microscope.

Interneurons were identified from their spiking responses to step-current pulses during the neurobiotin infusion as well as on the basis of morphological criteria after filling (dendrites with beaded appearance; absence of mushroom spines; absence of a prominent apical dendrite). The axonal arbors of 11 out of 49 neurobiotin-filled neurons (five cells in control conditions, and six cells in deprived conditions) showed high-contrast axonal labeling in L2/3 that could be traced back to the filled soma in L5B; these arbors were chosen for morphometric analysis. Ten L2/3 axon segments in each condition, ranging in length from 125 to 2,063 µm, were reconstructed manually using the freeware Neuromantic [56]. Varicosities were identified as focal swellings that appeared larger and brighter than the neighboring stretches of axon and could, due to their size and brightness and the axial resolution of the microscope, also be seen in at least two adjacent confocal image planes [57]. Objects with a maximum diameter of 2 µm were counted as single varicosites if no constriction of the circumference was evident; the rare objects whose maximum diameter exceeded 2 µm were counted as two varicosities.

### Data Analysis

Data were analyzed as described [5], using Igor 6 (Wavemetrics) and SPSS 17 (IBM). Briefly, maps of inhibitory inputs were constructed from electrophysiological signals recorded during 8–10 sweeps of the stimulation grid. IPSCs were identified by three criteria. First, the amplitude of the upward deflection in the averaged trace had to exceed 3 times the average standard deviation of current fluctuations in the absence of an optical stimulus (rms noise). Our conclusions are robust under different choices of threshold (2 and 4 rms noise). In exceptional cases, factors of 2.5–5 rms noise were applied to isolated maps to compensate for unusually low or high baseline activity in the recordings. Second, IPSCs had to reach half-maximal amplitude within 5–70 ms after optical stimulus onset. Third, IPSCs had to occur in at least three of the 8–10 sweeps and exhibit a temporal jitter of less than ±10 ms.

The presynaptic sources of IPSCs were allocated to individual cortical layers, which were identified by differences in shading and cell density [5],[20]–[22]. The strength of each synaptic input was measured by integrating the recorded current over a 100-ms interval, beginning at 5 ms before the rising IPSC reached its half-maximal amplitude; this measure thus represents the charge transfer per IPSC (Figure 9 and Figure 10). The contribution of a layer to the total amount of inhibition received by a target cell (Figure 3B and Figure 5B) was quantified as a percentage, which was obtained by calculating the product of the number of IPSCs originating from that layer and their average charge transfer (Figure 3C and Figure 5C) and normalizing this value to the total inhibitory charge flow of the cell. Differences between multiple experimental conditions were analyzed by one-way ANOVA, which was followed by pairwise Bonferroni-corrected *t* test. Pairwise hypotheses were evaluated by *t* test.

To visualize inhibitory input maps and their rearrangements following whisker trimming and regrowth, input strengths (the normalized charge flowing during 100 ms after IPSC onset) were coded in normalized gray scale (Figure 3A and Figure 5A; see Figure S2 for nonnormalized maps). For comparisons across experimental conditions, input maps were scaled and aligned to barrel septa in the horizontal dimension and to the L1–L2/3 and L5A–L5B borders in the vertical dimension (Figure 3A, Figure 5A, and Figure S2).

## Supporting Information

Figure S1 **Passive membrane properties of layer 2/3 pyramidal cells.** Membrane resistance (left) and membrane capacitance (right) of L2/3 pyramidal cells in barrel-related columns of mice with intact (black, *n* = 23) or trimmed (gray, *n* = 23) whiskers. Measurements used the membrane test routine built into pClamp10 (Axon Instruments) immediately after establishment of the whole-cell patch-configuration.
(TIF)

Figure S2 **Individual input maps.** Maps of inhibitory inputs to individual L2/3 pyramidal neurons in barrel-related columns representing intact (top), trimmed (second from top), or previously deprived whiskers after regrowth for 1 mo (second from bottom) or 3 mo (bottom). The amplitude of the IPSC evoked at each location is represented in gray scale, according to the look-up table at the bottom. The selected neurons represent deciles in the frequency distribution of the number of L5B-derived inputs to L2/3 pyramidal cells. Maps are scaled to the size of a standard whisker-related barrel (yellow outline).
(TIF)

Figure S3 **Variability of IPSC amplitudes.** Coefficients of variation of the individual IPSC amplitudes shown in Figure 9. IPSCs were evoked by optical stimulation of interneurons in the indicated cortical layers of barrel-related columns representing intact, trimmed, and previously deprived whiskers after regrowth for 1 mo and 3 mo.
(TIF)

Figure S4 **Distribution of IPSC amplitudes.** Histograms display the number of verified input sources (*x*-axis) of a given amount of charge transfer (*y*-axis) grouped in bins of 1 pC (layer 1–4) or 0.5 pC (layer 5). Data correspond to IPSC traces in Figure 9, and bars of average charge flow are shown for reference as displayed in Figure 9. Histograms are shown for inhibitory input sources of layer 1 to 5B (top to bottom) in barrel-related columns representing intact (top), trimmed (center-left), or previously deprived whiskers after regrowth for 1 mo (center-right) or 3 mo (right). Absolute number of locations in the indicated source layers (rows) giving rise to IPSCs of the indicated charges in L2/3 pyramidal neurons. Same data as in Figure 9 (barrel-related columns A, B, D, and E) and Figure 10 (barrel-related column C). Dark yellow columns representing average charge flow are reproduced for reference from Figure 9 and Figure 10.
(TIF)

Table S1 **Estimate of the number of interneurons activated per stimulation site.** The number of ChR2-expressing interneurons activated by a single optical pulse was calculated using two estimates of interneuron densities in specific layers of primary somatosensory cortex (S1) of the mouse [24],[25] and the empirically supported assumptions of a lateral optical resolution of ∼60 µm FWHM (Figure 1A and Figure 2E) [5], a response reliability of >90% (Figure 2B) [5], a slice thickness of 310 µm, and a scatter coefficient of ∼10 mm^−1^ [23].
(DOCX)

## Abbreviations

4-AP: 4-aminopyridine
aCSF: artificial cerebrospinal fluid
ANOVA: analysis of variance
ChR2: channelrhodopsin-2
DIC: differential interference contrast
FWHM: full width at half maximum
fs: fast-spiking
IPSC: inhibitory postsynaptic current
ISI: interspike interval
L: cortical layer
M1: primary motor cortex
NA: numerical aperture
non-fs: non-fast-spiking
PBS: phosphate-buffered saline
PFA: paraformaldehyde
POM: posterior medial nucleus
rms: root mean square
SD: standard deviation
S1: primary somatosensory cortex
TBS: Tris-buffered saline
TTX: tetrodotoxin
V1: primary visual cortex
