# Supplementary figures and images for: Experience-Dependent Rewiring of Specific Inhibitory Connections in Adult Neocortex
Source: PLoS Biol. 2014 Feb 25;12(2):e1001798. doi: 10.1371/journal.pbio.1001798 (PMC3934820; doi:10.1371/journal.pbio.1001798)

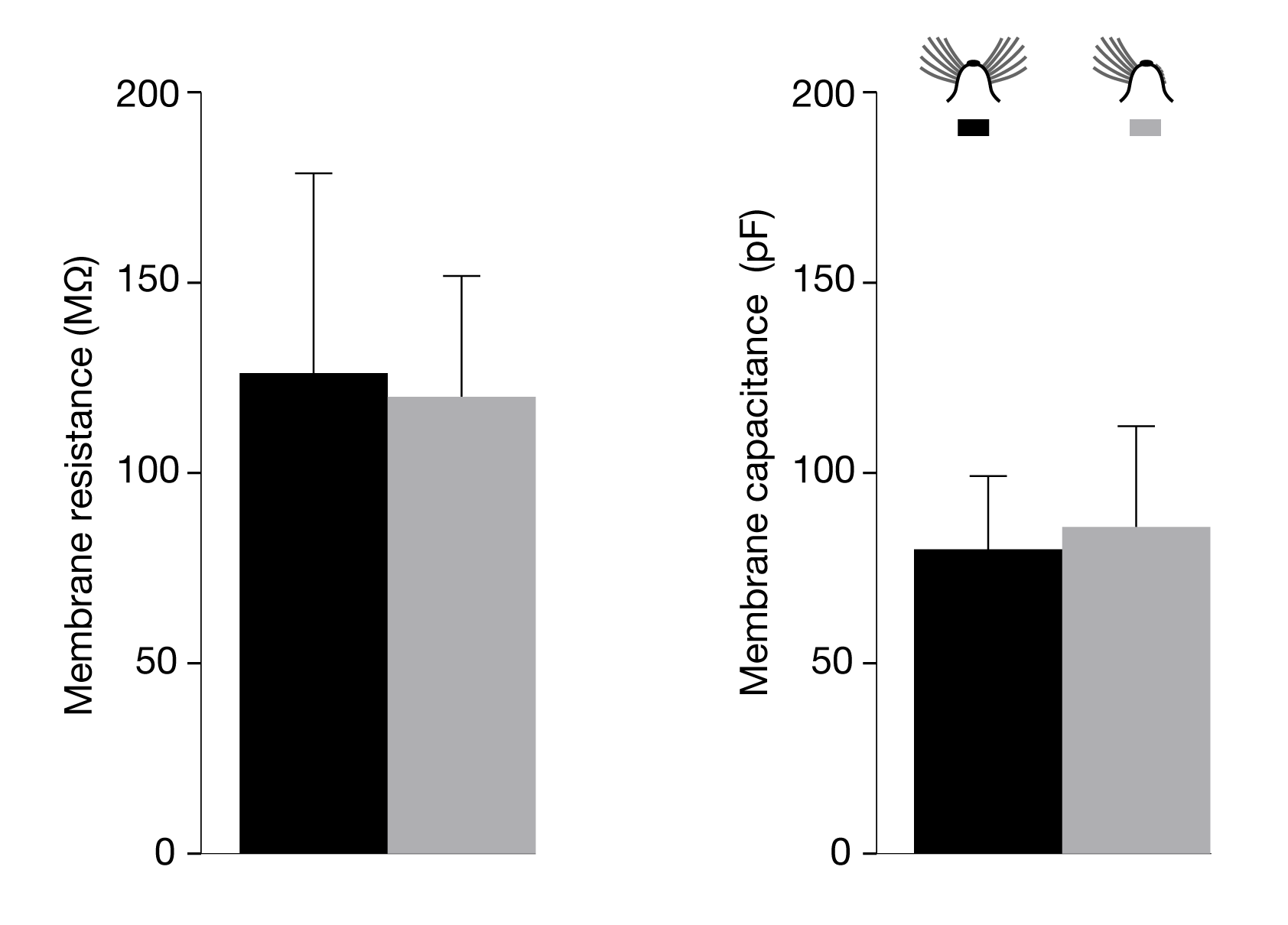

Supplement: Figure S1 — Passive membrane properties of layer 2/3 pyramidal cells. Membrane resistance (left) and membrane capacitance (right) of L2/3 pyramidal cells in barrel-related columns of mice with intact (black, n = 23) or trimmed (gray, n = 23) whiskers. Measurements used the membrane test routine built into pClamp10 (Axon Instruments) immediately after establishment of the whole-cell patch-configuration. (TIF) [file pbio.1001798.s001.tif]

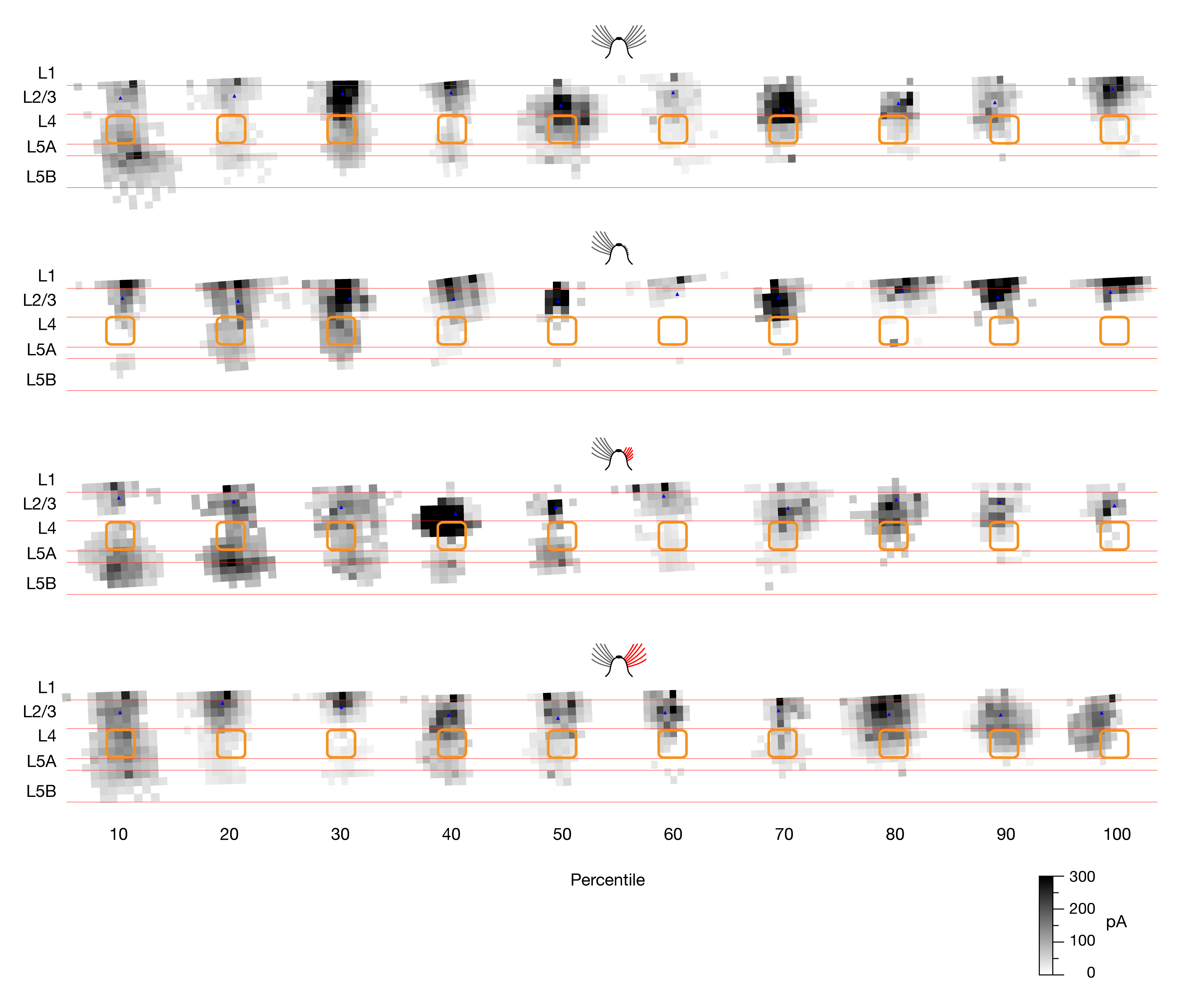

Supplement: Figure S2 — Individual input maps. Maps of inhibitory inputs to individual L2/3 pyramidal neurons in barrel-related columns representing intact (top), trimmed (second from top), or previously deprived whiskers after regrowth for 1 mo (second from bottom) or 3 mo (bottom). The amplitude of the IPSC evoked at each location is represented in gray scale, according to the look-up table at the bottom. The selected neurons represent deciles in the frequency distribution of the number of L5B-derived inputs to L2/3 pyramidal cells. Maps are scaled to the size of a standard whisker-related barrel (yellow outline). (TIF) [file pbio.1001798.s002.tif]

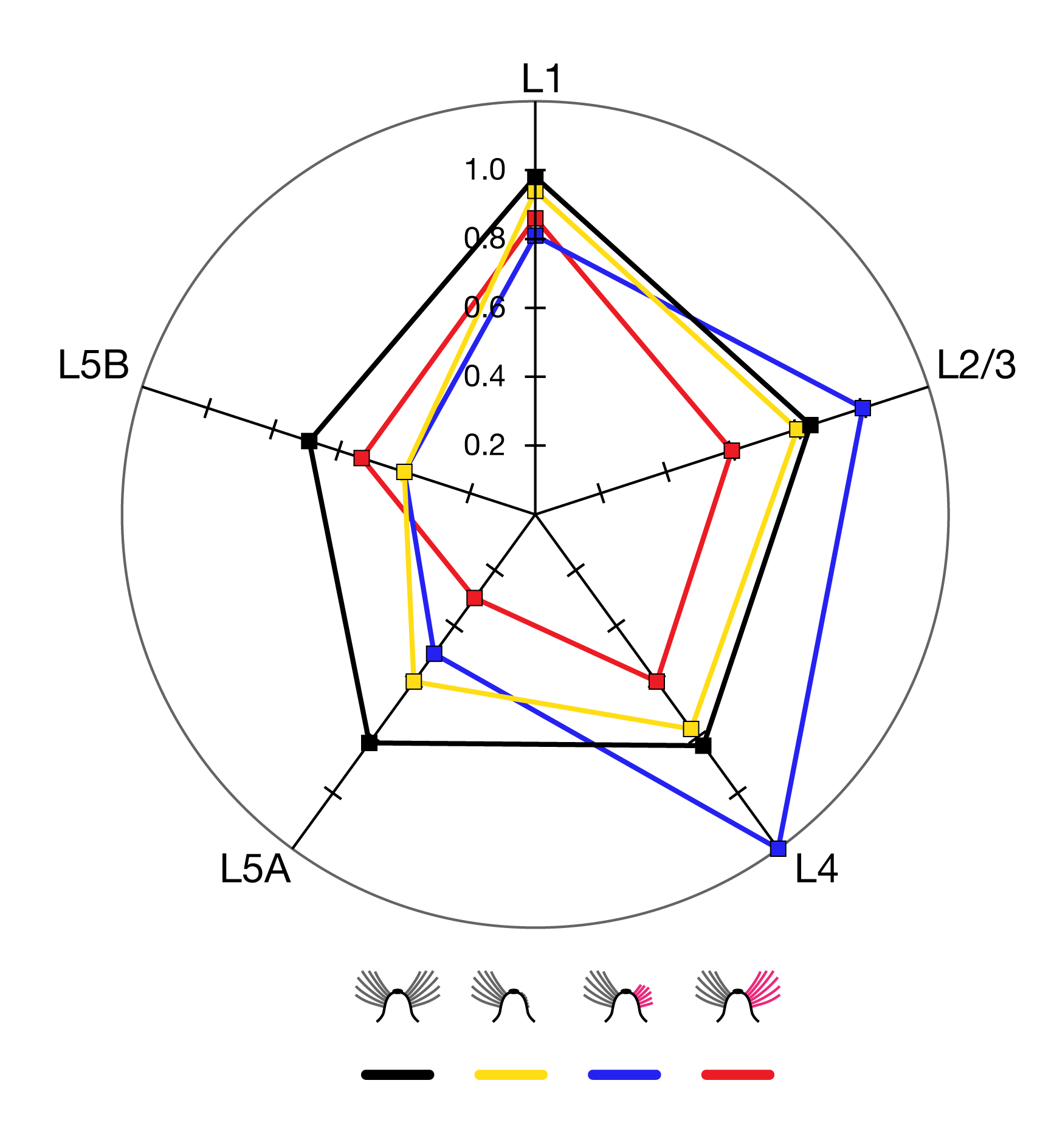

Supplement: Figure S3 — Variability of IPSC amplitudes. Coefficients of variation of the individual IPSC amplitudes shown in Figure 9. IPSCs were evoked by optical stimulation of interneurons in the indicated cortical layers of barrel-related columns representing intact, trimmed, and previously deprived whiskers after regrowth for 1 mo and 3 mo. (TIF) [file pbio.1001798.s003.tif]

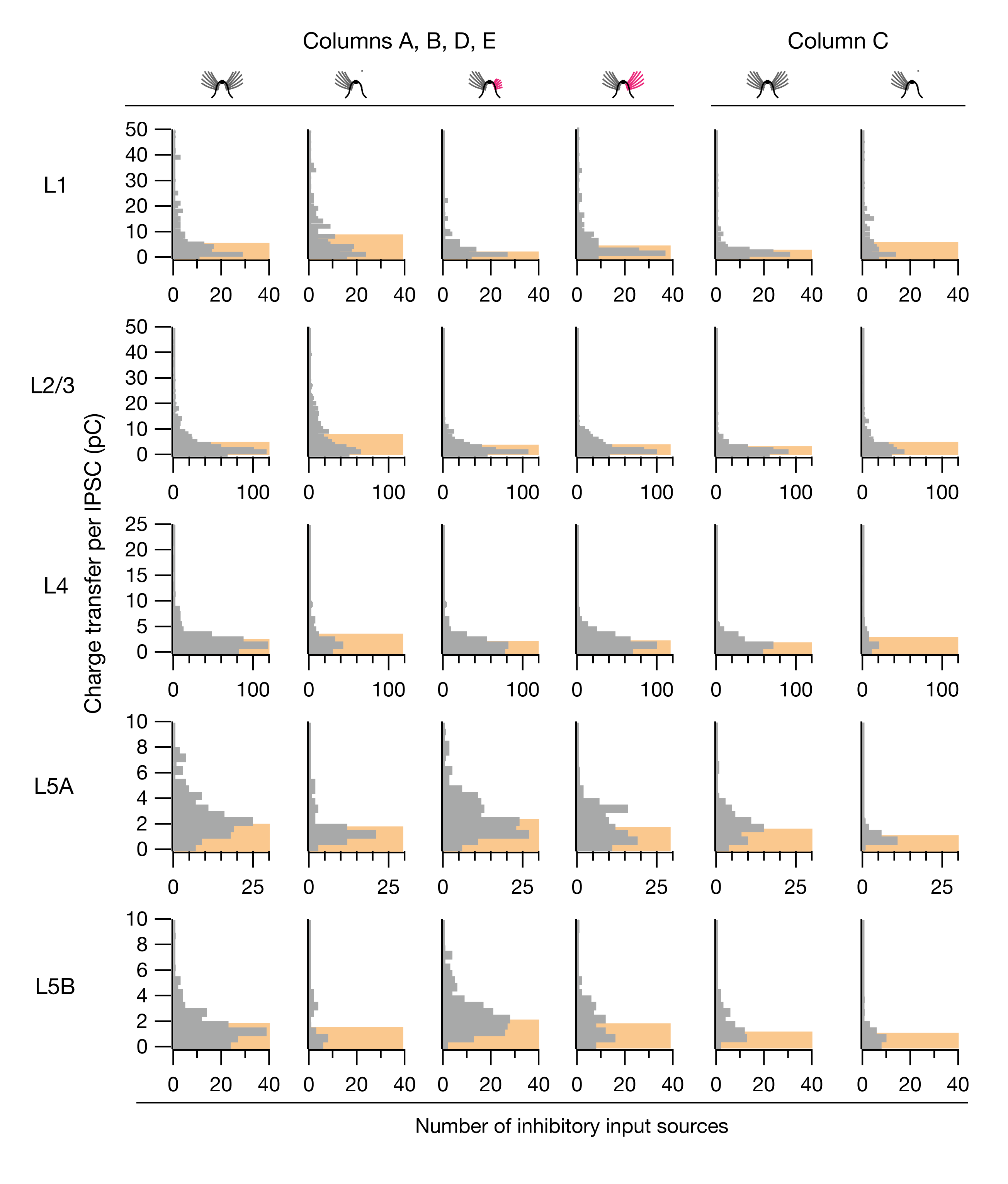

Supplement: Figure S4 — Distribution of IPSC amplitudes. Histograms display the number of verified input sources (x-axis) of a given amount of charge transfer (y-axis) grouped in bins of 1 pC (layer 1–4) or 0.5 pC (layer 5). Data correspond to IPSC traces in Figure 9, and bars of average charge flow are shown for reference as displayed in Figure 9. Histograms are shown for inhibitory input sources of layer 1 to 5B (top to bottom) in barrel-related columns representing intact (top), trimmed (center-left), or previously deprived whiskers after regrowth for 1 mo (center-right) or 3 mo (right). Absolute number of locations in the indicated source layers (rows) giving rise to IPSCs of the indicated charges in L2/3 pyramidal neurons. Same data as in Figure 9 (barrel-related columns A, B, D, and E) and Figure 10 (barrel-related column C). Dark yellow columns representing average charge flow are reproduced for reference from Figure 9 and Figure 10. (TIF) [file pbio.1001798.s004.tif]
