# Supplementary material for: Experience-Dependent Rewiring of Specific Inhibitory Connections in Adult Neocortex
Source: PLoS Biol. 2014 Feb 25;12(2):e1001798. doi: 10.1371/journal.pbio.1001798 (PMC3934820; doi:10.1371/journal.pbio.1001798)
Supplement: Table S1 — Estimate of the number of interneurons activated per stimulation site. The number of ChR2-expressing interneurons activated by a single optical pulse was calculated using two estimates of interneuron densities in specific layers of primary somatosensory cortex (S1) of the mouse [24],[25] and the empirically supported assumptions of a lateral optical resolution of ∼60 µm FWHM (Figure 1A and Figure 2E) [5], a response reliability of >90% (Figure 2B) [5], a slice thickness of 310 µm, and a scatter coefficient of ∼10 mm−1 [23]. (DOCX) [file pbio.1001798.s005.docx]

**Table S1. Estimate of the Number of Interneurons Activated per Stimulation Site**

|  | Number of interneurons activated per stimulation site | |
| --- | --- | --- |
| **Layer** | Lefort et al., 2009 | Xu et al., 2010 |
| L1 | 4.6 | 29.6 |
| L2/3 | 17.9 | 42.6 |
| L4 | 18.6 | 43.0 |
| L5A | 16.9 | 38.4 |
| L5B | 16.2 |  |
| L6 | 10.9 | 22.4 |
